# An estimate of asthma prevalence in Africa: a systematic analysis

**DOI:** 10.3325/cmj.2013.54.519

**Published:** 2013-12

**Authors:** Davies Adeloye, Kit Yee Chan, Igor Rudan, Harry Campbell

**Affiliations:** Centre for Population Health Sciences, University of Edinburgh Medical School, Edinburgh, UK

## Abstract

**Aim:**

To estimate and compare asthma prevalence in Africa in 1990, 2000, and 2010 in order to provide information that will help inform the planning of the public health response to the disease.

**Methods:**

We conducted a systematic search of Medline, EMBASE, and Global Health for studies on asthma published between 1990 and 2012. We included cross-sectional population based studies providing numerical estimates on the prevalence of asthma. We calculated weighted mean prevalence and applied an epidemiological model linking age with the prevalence of asthma. The UN population figures for Africa for 1990, 2000, and 2010 were used to estimate the cases of asthma, each for the respective year.

**Results:**

Our search returned 790 studies. We retained 45 studies that met our selection criteria. In Africa in 1990, we estimated 34.1 million asthma cases (12.1%; 95% confidence interval [CI] 7.2-16.9) among children <15 years, 64.9 million (11.8%; 95% CI 7.9-15.8) among people aged <45 years, and 74.4 million (11.7%; 95% CI 8.2-15.3) in the total population. In 2000, we estimated 41.3 million cases (12.9%; 95% CI 8.7-17.0) among children <15 years, 82.4 million (12.5%; 95% CI 5.9-19.1) among people aged <45 years, and 94.8 million (12.0%; 95% CI 5.0-18.8) in the total population. This increased to 49.7 million (13.9%; 95% CI 9.6-18.3) among children <15 years, 102.9 million (13.8%; 95% CI 6.2-21.4) among people aged <45 years, and 119.3 million (12.8%; 95% CI 8.2-17.1) in the total population in 2010. There were no significant differences between asthma prevalence in studies which ascertained cases by written and video questionnaires. Crude prevalences of asthma were, however, consistently higher among urban than rural dwellers.

**Conclusion:**

Our findings suggest an increasing prevalence of asthma in Africa over the past two decades. Due to the paucity of data, we believe that the true prevalence of asthma may still be under-estimated. There is a need for national governments in Africa to consider the implications of this increasing disease burden and to investigate the relative importance of underlying risk factors such as rising urbanization and population aging in their policy and health planning responses to this challenge.

Chronic respiratory diseases (CRDs) are among the leading causes of death worldwide, with asthma rated the most common chronic disease affecting children (1). Globally, about 300 million people have asthma, and current trends suggest that an additional 100 million people may be living with asthma by 2025 (1,2). The World Health Organization (WHO) estimates about 250 000 deaths from asthma every year, mainly in low- and middle-income countries (LMIC) (3,4). Just like with many other chronic diseases in Africa, the fast rate of urbanization has been linked to the increase in the burden of asthma and other allergic diseases (3,5,6). The prevalence of these conditions may, in theory, have the potential to reach levels higher than those observed in high-income countries (HIC) due to priming effects of parasitic helminthic infections on the immune system, as these infections are common in many African settings (5). The International Study of Asthma and Allergies (ISAAC) reported that asthma prevalence among children was increasing in Africa and has contributed most to the burden of disease through its effects on quality of life (3). In-patient admissions and purchase of medications account for most of the direct costs on government, while loss of productivity, due to absenteeism from work and school, are responsible for most of the indirect costs (7,8).

Asthma is widely known as a multifactorial respiratory disorder with both genetic and environmental underlying risk factors (3). Exposure to common allergens (including pollens, dust mites, and animal furs) and indoor and outdoor air pollution from various sources (eg, traffic pollution, combustion of fossils and biomass fuels, workplace dust) have all been implicated as triggers of the disease (9). Second hand tobacco smoking is a confirmed risk factor in pediatric patients (5,10). Viral infections, a major cause of upper respiratory tract infections and “common cold,” are also a common risk factor in children (11,12). As noted, helminthic infections are relatively common in Africa and are associated with bronchial hyper-responsiveness and asthma (5,13); this is perhaps due to the presence of related raised immunoglobulin E (IgE) and a prominent Th2 immune response (5,14).

Studies on asthma are few in Africa, with most publications mainly from South African and Nigerian populations (14). One main factor affecting research output is the diagnosis of asthma, which still remains a challenging issue (15,16). The WHO has emphasized that this has limited on-going research efforts globally (4,16). The International Union against Tuberculosis and Lung Diseases (IUATLD) published one of the first diagnostic and survey guidelines for asthma in 1984, but experts subsequently reported concerns about its precision and reliability (17). According to the Global Initiative for Asthma (GINA), detailed history, physical examination and spirometric lung function tests are vital to the diagnosis and management of asthma (10,18). Generally, a reduction in forced expiratory volume in one second (FEV_1_) and peak expiratory flow (PEF) may be indicative of asthma, with the amount of reduction proportional to the severity of asthma (4). GINA proposed that an increase in FEV_1_ of >12% and 200 mL in about 15-20 minutes following the inhalation of 200-400 μg of salbutamol or a 20% increase in PEF from baseline can be employed as standardized criteria in diagnosis of asthma (10). This, however, lacks sensitivity, as many asthmatics, especially those on treatment, may not exhibit an increase in FEV_1_ and PEF when assessed (16,19). Thus, although asthma is characterized by significant reversibility of airway obstruction, an absence of reversibility may not always exclude the presence of asthma (20). The ISAAC established in 1991, remains the largest epidemiological study among children globally (1). ISAAC methodologies and scoring are currently the most widely employed by researchers in Africa (1,4). This involves both video and written questionnaires, as there were reports that video and pictorial representations of asthma symptoms may contribute to improved case recognition in younger children (1). However, this is still a subject of debate among experts (21). The European Community Respiratory Health Survey (ECRHS), which assessed the prevalence of atopy and symptoms of airway disease among older age groups in Western Europe, has been widely implemented and has reported significant geographic variations in the prevalence of asthma and atopy (9). Despite these revised guidelines, both ISAAC and ECRHS research groups have reported challenges in achieving high sensitivity and specificity in case ascertainment with the symptom “wheeze at rest in the last 12 months” (also regarded as current wheeze, or active wheeze), yielding the highest sensitivity and specificity (1).

In Africa, problems including those arising from the over-utilization of health services, lack of trained staff and diagnostic apparatus, and non-availability and unaffordability of inhaled medications have hindered efforts to improve the management of asthma (22,23). The lack of organized health promotion programs, such as effective control strategies for environmental triggers, air pollutants, and occupational dusts have also contributed to the growing burden (24). The WHO has reported that the levels of asthma control and health responses in the continent have been below recommended standards, and that these have contributed to the size of the disease burden (3,4). In addition, although many African countries have national guidelines for the management of asthma and other CRDs, these guidelines have not been implemented in most rural areas (25,26). Economic analyses in many African settings have shown that direct costs from asthma are usually greater than the indirect costs. However, indirect costs represent a relatively higher proportion of total costs among pediatric than adult patients (8). Moreover, the wider economic burden on individuals, families, employers, and society, due to loss of future potential source of livelihood, has also been devastating in many resource-poor settings (22). It is believed that many children with asthma in Africa may fail to achieve their full potential if proper management and control measures are not put in place (1). It has been suggested that education of health care providers and the public is a vital element of the response to the challenge posed by asthma in Africa (4,27).

By 2015, it is expected that world’s urban population will increase from 45% to 59%, with over half of this occurring in Africa (8). It is also expected that the prevalence of asthma and many chronic diseases in Africa will increase due to this growing population size and from effects of accompanying urbanization and adoption of western lifestyles (28). In light of this and of the low research output and poor availability of health services data on the burden of asthma in Africa, it is important to analyze the available data through a systematic review of the literature in order to attempt to quantify the burden, guide health priority settings, and inform the formulation of an appropriate health policy response.

## METHODS

### Search strategy and selection criteria

We conducted a systematic search of Medline, EMBASE, and Global Health. After an initial scoping exercise to identify Medical Subject Headings (MESH) and keywords, we developed a final search strategy. We further hand-searched reference lists of retained publications for more relevant studies. African countries were included as contained in the World Bank list of economies from July 2012 (29) (Table 1**)**.

**Table 1:** Search terms used in the study

1	africa/ or exp africa, northern/ or exp algeria/ or exp egypt/ or exp libya/ or exp morocco/ or exp tunisia/ or exp “africa south of the sahara”/ or exp africa, central/ or exp cameroon/ or exp central african republic/ or exp chad/ or exp congo/ or exp “democratic republic of the congo”/ or exp equatorial guinea/ or exp gabon/ or exp africa, eastern/ or exp burundi/ or exp djibouti/ or exp eritrea/ or exp ethiopia/ or exp kenya/ or exp rwanda/ or exp somalia/ or exp sudan/ or exp tanzania/ or exp uganda/ or exp africa, southern/ or exp angola/ or exp botswana/ or exp lesotho/ or exp malawi/ or exp mozambique/ or exp namibia/ or exp south africa/ or exp swaziland/ or exp zambia/ or exp zimbabwe/ or exp africa, western/ or exp benin/ or exp burkina faso/ or exp cape verde/ or exp cote d'ivoire/ or exp gambia/ or exp ghana/ or exp guinea/ or exp guinea-bissau/ or exp liberia/ or exp mali/ or exp mauritania/ or exp niger/ or exp nigeria/ or exp senegal/ or exp sierra leone/ or exp togo/
2	exp vital statistics/ or exp incidence/
3	(incidence* or prevalence* or morbidity or mortality).tw.
4	(disease adj3 burden).tw.
5	exp “cost of illness”/
6	exp quality-adjusted life years/
7	QALY.tw.
8	Disability adjusted life years.mp.
9	(initial adj2 burden).tw.
10	exp risk factors/
11	2 or 3 or 4 or 5 or 6 or 7 or 8 or 9 or 10
12	exp asthma/ or exp asthma, aspirin-induced/ or exp asthma, exercise-induced/ or exp asthma, occupational/ or exp status asthmaticus/
13	1 and 11 and 12

We retained cross-sectional population-based studies on asthma conducted primarily on African population groups. The date of publication was set from 1990 to June 2013. We included studies providing numerical estimates on the prevalence of asthma, including non-English publications. We excluded studies that were mainly reviews, hospital-based (without a denominator population estimate), without numerical estimates, and conducted on non-human subjects.

Studies were further checked for clear diagnostic criteria and survey methods. As noted above, asthma has been described in various ways by many researchers, we therefore allowed for these variations in our analysis (Table 2 and Supplementary Table 1 (supplementary table 1)). However, most survey methods were mainly based on written or video questionnaires, as proposed by the ISAAC study group (Table 3 and Supplementary Table 1 (supplementary table 1)).

**Table 2:** Asthma terms and definitions

Terms	Definition
Wheeze	A high pitched whistling sound originating from obstructed airways (1). “Wheeze at rest-12 months” refers to the prevalence of wheeze in a person in the last 12 mo.
Asthma	A chronic airway disease characterized by wheezing (a high pitched whistling sound originating from obstructed airways). Patient usually presents with chronic airways inflammation, bronchial hyper-responsiveness and reversible airflow obstruction, resulting in the recurrent attacks of wheeze, chest tightness, breathlessness, and occasionally cough and sputum production, all of varying severity and frequency from person to person (1,3,24,87). Asthma ever refers to cummulative prevalence of asthma in a person.
Asthma exacerbation	Also known as acute asthma. A sudden progressive episodes of shortness of breath, usually characterized by chest tightness, wheezing, cough, or sputum production (87)
Moderate asthma exacerbation	An event that, when recognized, should result in a temporary change in treatment, in an effort to prevent the exacerbation from being severe (24)
Severe asthma exacerbation	Events that require urgent action on the part of the patient and physician to prevent a serious outcome, such as hospitalization or death (24)
Severe asthma	Uncontrolled asthma which can result in risk of frequent severe exacerbations (or death), and/or adverse reactions to medications, and/or chronic morbidity, including impaired lung function or reduced lung growth in children (24)
Asthma control	Extent to which the various manifestation of asthma are reduced or removed by treatment (3,24)
Asthma diagnosis	GINA proposed a holistic approach involving detailed history, physical examination and spirometry. An increase in FEV_1_ of ≥12% and ≥200ml after a bronchodilator is indicative of reversible airflow limitation, which is consistent with asthma. Peak expiratory flow (PEF) with an improvement of 60l/min (or ≥20% of the pre-bronchodilator PEF) after a bronchodilator, or a diurnal variation in PEF of more than 20% (with twice daily readings more than 10%) may also be indicative of asthma. Other non-specific diagnostic tests include methacholine or histamine test, inhaled mannitol or exercise challenge, skin prick test and measurement of serum IgE (10,18)

^*Abbreviations: FEV1 – forced expiratory volume in one second; GINA – Global Initiative for Asthma; IgE – immunoglobulin E; PEF – peak expiratory flow.^

**Table 3:** Asthma study distribution

Country:	Number of studies
Algeria (32,36,37,44)	4
Burkina Faso (55,56)	2
Cameroon (31)	1
Cape Verde (72)	1
Congo Brazzaville (31)	1
Cote d’Ivoire (31,47,63)	3
DR Congo (31,61)	2
Egypt (52)	1
Ethiopia (12,31,32,53,71,73)	6
Gabon (31)	1
Gambia (69)	1
Ghana (41)	1
Guinea (31)	1
Kenya (31,39,40,59,68)	5
Madagascar (66)	1
Morocco (32,34,44,48)	4
Mozambique (49)	1
Nigeria (31-33,38,45,50,51,58)	8
Rwanda (57)	1
South Africa (20,31,32,42,43,54,60,62,65,67,70)	11
Sudan (31,32)	2
Tanzania (35)	1
Togo (31)	1
Tunisia (32,44,46,64)	4
**Duration of study:**	
<1 y	23
1-3 y	16
>3 y	6
**Sample size:**	
<1000	18
1001-3000	15
>3000	12
**Study setting:**	
rural	3
urban	6
mixed	30
occupational	8
**Study type:**	
based on ISAAC guidelines*	14
non-ISAAC guidelines	31
written questionnaire	39
video questionnaire	6

*ISAAC – The International Study of Asthma and Allergies.

### Data extraction and analysis

Relevant data were extracted from retained studies and saved in Microsoft Excel file-format. All data were double extracted and sorted by country, study period, age, and their respective case number, sample size, and prevalence estimate. Extracts were grouped into data from written questionnaires or video questionnaires, both including data based on asthma diagnosis, and/or its symptoms (wheeze at rest, wheeze on exercise, nocturnal wheeze, nocturnal cough, or severe wheeze). For studies conducted on the same study site, population or cohort, the first chronologically published study was selected, with all additional data from other studies added to that of the selected paper.

For our analysis, weighted means of asthma symptoms were calculated (pooled from reported crude prevalences in individual studies) and expressed in percentages. Asthma prevalence estimates based on “current wheeze” (wheeze at rest- 12 months) have high sensitivities and specificities (1,17,30); we therefore applied extracted values from this in our modeling. Mean age and prevalence were plotted on bubble graphs, and the fitted curve explaining the largest proportion of variance (best fit) was applied. The equation generated determined the prevalence of asthma in Africa at midpoints of the United Nation population 5-year age-groups (for Africa) for the years 1990, 2000, and 2010.

## RESULTS

### Systematic review

**Figure 1:**
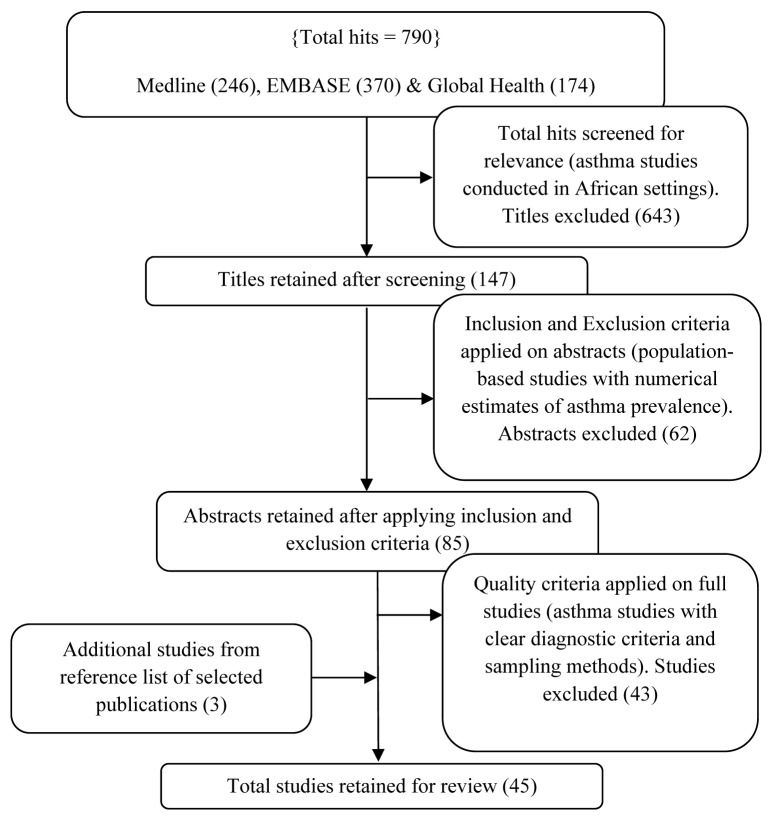
Search strategy.

Our main search returned 790 studies; Medline (n = 246), EMBASE (n = 370), and Global Health (n = 174). After screening the titles for relevance (ie, asthma studies conducted primarily in an African population setting) and excluding duplicates, 147 studies were retained. 85 abstracts satisfied our selection criteria (ie, population-based studies providing numerical prevalence estimates of asthma). Applying the quality criteria (ie, studies with clear diagnostic criteria and survey methods), 43 studies were excluded. On hand searching reference lists of selected studies, further 3 studies were included, giving a total of 45 studies retained for the review (Figure 1).

### Study characteristics

The 45 retained studies (12,20,31-73) covered most parts of Africa. South Africa (11 studies) and Nigeria (8 studies) had the highest publication outputs. Ethiopia and Kenya closely followed with 6 and 5 studies respectively, while Algeria, Morocco, and Tunisia in North Africa had 4 studies each. 14 studies were based on ISAAC guidelines and 31 non-ISAAC guidelines, while 39 studies employed written questionnaires and 6 used video questionnaires (Table 3).

Most studies were conducted mainly on pediatric subjects, mostly defined as less than 15 years of age. For the age determination of subjects from most studies, birth certificates were usually employed, and in the absence of valid age-verification documents, historical landmarks were employed.

Many studies were conducted within one year (23 studies), sample sizes were mostly less than 1000 (mean 3243; median 2067), and study settings were predominantly mixed (urban and rural) (36 studies) (Table 3).

### Prevalence estimates

We observed that the prevalence of asthma in some parts of Africa was comparable with that reported from surveys in high-income settings. From studies based on written questionnaires, “asthma ever” (cumulative prevalence of asthma) was highest in South Africa (53%, 5-12 years) in 1997 (20), followed by Egypt (26.5%, 11-15 years) in 2005 (52), Nigeria (18.4%, 15-35 years) in 1995 (45), and Ethiopia (16.3%, >20 years) in 1997 (73). The lowest prevalence was recorded in Gambia (1.9%, >15 years) in 1997 (69). “Current wheeze” (wheeze at rest-12-months) was consistently high in South Africa, 26.8% (13-14 years) in 1994, 23.9% (5-12 years) in 1998, and 20.3% (13-14 years) in 2003 (20,31,62). From studies based on video questionnaires, “current wheeze” was highest in Morocco (12.9%, 6-7 years) in 2003 (31) and Tanzania (12.3%, 9-10 years) in 2008 (35), with South Africa recording the lowest prevalence (6.5%, 6-7 years) in 1995 and 2000, respectively (31,62); there was no reported prevalence of “asthma ever” from studies based on video questionnaires. However, from all studies, the pooled crude prevalences (weighted means) for “current wheeze” was 13.2% (male 10.8%, female 13.1%, mean age 18.4 years), and “asthma ever” was 6.6% (male 6.7%, female 6.3%, mean age 22.9 years) (Table 4**)**. We observed that the pooled crude prevalences were consistently higher among urban dwellers than rural dwellers. “Current wheeze” was 9.6% (male 12.1%, female 7.0%, mean age 19.6 years) in urban settings and 7.0% (male 5.5%, female 3.8%, mean age 17.5 years) in rural settings. “Asthma ever” prevalence was 5.9% (male 5.6%, female 3.9%, mean age 22.9 years) and 5.1% (male 4.2%, female 3.1%, mean age 17.5 years) in urban and rural dwellers, respectively (Table 5).

**Table 4:** Weighted mean prevalence (pooled crude prevalences) of asthma symptoms from all studies

Study type	Asthma indices	Overall study characteristics		All		Male		Female	
		study period	mean age (years)	data points	weighted mean % (95% confidence interval)	data points	weighted mean % (95% confidence interval)	data points	weighted mean % (95% confidence interval)
**Written questionnaire**	Asthma (ever)	1993-2008	22.9	50	6.6 (4.7-8.6)	6	6.7 (2.3-11.3)	6	6.3 (2.3-10.1)
	Wheeze at rest (12 mo)	1993-2010	18.4	46	13.2 (11.6-14.9)	7	10.8 (5.9-15.8)	7	13.1 (7.6-18.4)
	Wheeze at rest (ever)	1993-2010	14.7	12	17.7 (10.7-24.6)	2	9.1 (8.8-9.4)	2	6.2 (4.8-7.5)
	Wheeze after exercise (12 mo)	1995-2003	12.3	6	23.8 (15.9-31.7)	3	14.7 (4.4-33.8)	3	24.6 (11.0-38.2)
	Wheeze after exercise (ever)	1993-2010	12.0	11	8.9 (5.1-12.8)	1	6.5	1	6.5
	Nocturnal wheeze (12 mo)	1995-2005	15.4	6	4.9 (2.4-7.4)	1	9.1	1	10.0
	Nocturnal wheeze (ever)	-	-	-	-	-	-	-	-
	Nocturnal cough (12 mo)	1995-2005	14.1	7	24.2 (18.3-30.0)	3	24.5 (11.5-37.4)	3	28.1 (13.2-43.2)
	Nocturnal cough (ever)	1993	10.8	1	9.3	1	10.3	1	8.5
	Severe wheeze (12 mo)	1993-2003	13.1	25	4.7 (3.7-5.6)	4	6.0 (4.6-7.4)	4	3.0 (0.8-5.2)
	Severe wheeze (ever)	1993-2005	12.2	2	8.8 (5.9-11.6)	1	7.2	1	5.2
**Video questionnaire**	Wheeze at rest (12 mo)	1995-2008	13.0	9	9.7 (8.9-10.5)	1	11.7	1	13.0
	Wheeze at rest (ever)	1995-2008	13.1	8	13.2 (12.4-14.0)	1	16	1	18.2
	Wheeze after exercise (12 mo)	1995-2008	12.9	8	15.5 (14.7-16.3)	1	19.1	1	11.5
	Wheeze after exercise (ever)	1995-2008	13.0	7	20.4 (16.9-23.8)	1	28.7	1	15.4
	Nocturnal wheeze (12 mo)	1995-2008	13.0	8	5.6 (4.8-6.4)	1	9.6	1	5.1
	Nocturnal wheeze (ever)	1995-2008	13.0	8	8.1 (7.3-8.9)	1	14.9	1	19.2
	Nocturnal cough (12 mo)	1995-2008	12.9	8	17.7 (16.8-18.5)	1	22.3	1	20.5
	Nocturnal cough (ever)	1995-2008	13.0	7	24.6 (20.5-28.7)	1	31.9	1	34.6
	Severe wheeze (12 mo)	1995-2008	13.0	9	6.8 (6.1-7.6)	1	5.3	1	3.9
	Severe wheeze (ever)	1995-2008	13.1	8	9.6 (8.7-10.3)	1	12.8	1	13.0

**Table 5:** Rural and urban differences in asthma prevalences

Asthma indices	Study setting	Study characteristics		All		Male		Female	
**study period**	**mean age**	**data points**	**weighted mean % (95% confidence interval)**	**data points**	**weighted mean % (95% confidence interval)**	**data points**	**weighted mean % (95% confidence interval)**
Asthma (ever)	Rural	1993-2008	17.5	6	5.1 (7.2-9.4)	2	4.2 (2.1-6.3)	2	3.1 (0.8-6.2)
	Urban	1993-2010	22.9	7	5.9 (2.9-7.9)	1	5.6	1	3.9
Wheeze at rest (12 mo)	Rural	1993-2008	17.5	6	7.0 (2.5-11.5)	2	5.5 (1.4-12.4)	2	3.8 (1.5-12.9)
	Urban	1993-2005	19.6	7	9.6 (3.9-15.2)	1	12.1	1	7

**Figure 2:**
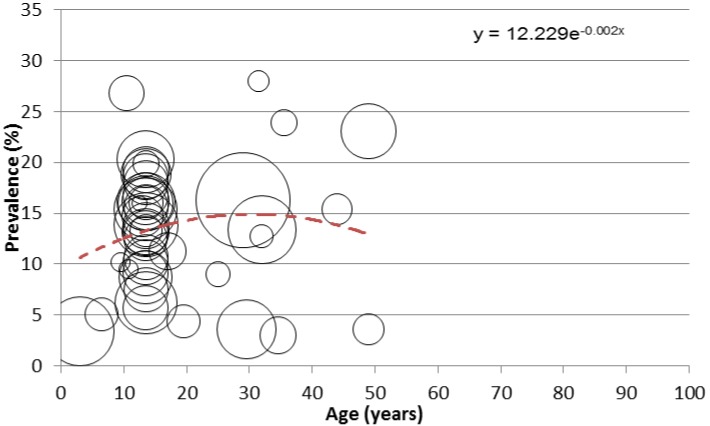
Age and prevalence distribution of asthma.

Our modeling revealed an increasing prevalence of asthma in Africa. We reported 34.1 million asthma cases (12.1%; 95% confidence interval [CI] 7.2-16.9) among children <15 years, 64.9 million asthma cases (11.8%; 95% CI 7.9-15.8) among people aged <45 years, and 74.4 million cases (11.7%; 95% CI 8.2-15.3) in the total population in 1990. This increased to 41.3 million (12.9%; 95% CI 8.7-17.0) among children <15 years, 82.4 million (12.5%; 95% CI 5.9-19.1) among people aged <45 years, and 94.8 million (12.0%; 95% CI 5.0-18.8) in the total population in 2000. In 2010, we estimated a total of 49.7 million asthma cases (13.9%; 95% CI 9.6-18.3) among children <15 years, 102.9 million (13.8%; 95% CI 6.2-21.4) among people aged <45 years, and 119.3 million cases (12.8%; 95% CI 8.2-17.1) in the total population. We could not model the asthma prevalences separately for men and women due to very limited data (Figure 2 and Table 6).

**Table 6:** Estimated asthma cases and prevalences based on current wheeze “wheeze at rest- 12 months” (based on bubble graph and United Nations population estimates for Africa)

Age range(years)	1990 estimates (000)	2000 estimates (000)	2010 estimates (000)
0-4	13405.19	15750.21	18946.71
5-9	11202.33	13526.59	16412.34
10-14	9461.50	12041.26	14306.87
15-19	7889.99	10494.98	12758.85
20-24	6497.60	8806.244	11373.60
25-29	5391.37	7149.79	9722.56
30-34	4457.30	5804.15	7946.30
35-39	3648.13	4823.34	6315.71
40-44	2977.33	3998.62	5075.31
45-49	2409.99	3260.32	4208.92
50-54	2009.40	2627.89	3490.57
55-59	1637.07	2064.60	2801.72
60-64	1282.71	1638.11	2172.14
65-69	919.99	1226.50	1581.25
70+ years	1162.36	1593.05	2201.80
**Asthma cases (<15 y)**	34069.02	41318.06	49665.92
**Estimated prevalence (<15 y)**	0.121 (95%CI: 0.072-0.169)	0.129 (95%CI: 0.088-0.170)	0.139 (95%CI: 0.096-0.183)
**Asthma cases (<45 y)**	64930.74	82395.18	102858.20
**Estimated prevalence (<45 y)**	0.118 (0.079- 0.158)*	0.125 (0.059- 0.191)	0.138 (0.062- 0.210)
**Asthma cases (all)**	74352.26	94805.65	119314.60
**Estimated prevalence (all)**	0.117 (0.082- 0.153)	0.119 (0.050- 0.188)	0.128 (0.082- 0.171)

*CI – confidence interval.

## DISCUSSION

This study, to our knowledge, provides the first systematic, data-derived and continent-wide estimates of asthma prevalence in Africa. Our modeling was based on published epidemiological data on “current wheeze” prevalence, which has relatively higher sensitivity and specificity (1,17,30), and also shows a significant association between age and asthma prevalence (74).

From the 2010 Institute for Health Metrics and Evaluation (IHME) global burden of disease estimates, chronic respiratory diseases overall burden has been decreasing globally and was responsible for about 4.7% of global disability adjusted life years (DALYs) in 2010, with asthma accounting for about one-fifth of this (75). However, the IHME reported that DALYs from asthma increased by 4.6% (from 21.5 to 22.5 million) between 1990 and 2010, while deaths decreased by 9.1% (from 0.38 to 0.34 million) over the same period globally (75,76). Concerns have been raised about the application of non-user friendly analytical methods, heavy statistical modeling, and difficulties in assessing the design methodologies and metrics used (77,78). In Africa however, the ISAAC study group, which mainly conducted epidemiological studies on asthma, reported increasing prevalences of asthma across many study settings (31). For example, the prevalence of “current wheeze” (wheeze at rest-12 months) among children aged 13-14 years old in South Africa increased from 16.1% to 20.3% between 1995 and 2002; Nigeria (West Africa) recorded an increase from 10.9% to 13.0%, Ethiopia (Horn of Africa) reported an increase from 6.2% to 9.1%, and Kenya (East Africa) an increase from 13.9% to 18.0% (31,32). Our pooled crude prevalences (weighted means) also reflect this increase; we reported a prevalence of current wheeze (wheeze at rest- 12-months) of 13.2% for both written and video questionnaires respectively. Furthermore, in keeping with findings from many studies, our pooled crude prevalences were consistently higher in urban than rural settings, suggesting the effects of increasing urbanization on asthma prevalence in Africa (68,69,71).

From our modeling, we estimated a prevalence of 11.7% for asthma, totaling over 74 million people in 1990, and our 2010 prevalence was 12.8%, about 120 million people. Public health experts have reported that increasing tobacco smoking without appropriate legislation and implementation of relevant health promotional measures in many LMIC, especially in Africa, may also be responsible for the increase in asthma and other chronic respiratory diseases’ burden in the region (2,75,79). In addition, the Global Burden of Asthma Report (GBAR) reported an increasing trend of asthma globally (4). GBAR estimated over 235 million asthma cases worldwide, and about 50 million people living with asthma in Africa in 2004, with the highest prevalence (8.1%) recorded in South Africa (4). The authors argue that this increasing trend is expected due to rise in atopic sensitizations, allergic conditions, and changing patterns of environmental triggers (associated with environmental smoking exposure in children, population growth, and urbanization) in Africa over the last two decades (4). These factors may therefore be indicative of our reported high estimates.

While we aimed to provide an improved prevalence estimate of asthma in Africa by carefully selecting high quality studies and applying simple analytical tools, there are however factors that could have affected our analysis. We thus entertain some degrees of uncertainties beyond the statistical confidence intervals generated, as variations in population settings, diagnostic criteria, sampling strategies, and effects of other health determinants (beyond age of patients) are factors that need be considered. First, the variation in diagnostic criteria was observed across many study settings, with criteria based on asthma symptoms and ISAAC scores frequently used. This could have reflected in our reported high estimates of asthma in Africa, as there is evidence suggesting the ISAAC studies could have over-estimated the prevalence of asthma in Africa, as most study centers were mainly urban (1); and with ISAAC studies conducted mainly in the age range 13-14 years, it may still not be representative of all age-groups and the overall population (1,15). Second, while many studies were cross-sectional population-based, we also included studies conducted in occupational settings; this is in view of reports revealing that occupational asthma contributes significantly to the global burden of chronic respiratory diseases (73,80), and as these studies reported high prevalences, we understand this could have increased our estimates, too. Third, despite included studies spreading across most parts of Africa (24 countries in total), there are still many countries in Africa that are not included in the review; this reflects data gaps in the continent, and thus the generalizability of our estimates for Africa may need to be carefully examined. In addition, our overall sample size of 187 904 (from all studies) may not be a representative sample of the general African population as there were more younger age groups. Finally, data on age- and sex-specific prevalence, including corresponding data on urban-rural settings, which are vital comparative indices in any study, were not always provided across many studies.

### Management of asthma and public health challenges in Africa

It is important to note that chronic respiratory disease burden, including asthma has continued to increase in Africa due to lack of appropriate response from the governments of many African countries (81,82). The national emphasis on asthma and relevant health messages have been sub-optimal, leading, in sequence, to poor awareness of the burden, low prioritization, inadequate staffing and resources, and very low budget allocation. In fact, budget allocation in many African countries mainly targets infectious diseases; funds have been greatly biased toward HIV/AIDS, malaria, and tuberculosis, as these are the main government priorities (28,83). The GBAR authors reported that poor government allocation of funds for asthma remained an important factor responsible for limited access to asthma medications, emergency health care, and other related health services in Africa (4). In addition, with tobacco companies still supporting many African governments, tobacco products’ sales have increased, and government funds have remained unavailable for research on asthma, as researches aimed at improving management of asthma may be against tobacco sales and consumption (22,69). This has greatly resulted in increased smoking (without a counter legislation) and a growing burden of asthma, especially among children in Africa over the last two decades (5,10). For example, Mackay et al reported that a comprehensive smoke free-legislation was important to achieving reduction in the asthma incidence among people without occupational exposure to environmental tobacco smoke; a reduction of 18.2% per year was observed in hospital admissions in Scotland among children <15 years in 2009 compared to a mean increase of admission of 5.2% per year before implementation of the legislation in 2006 (84)

The diagnosis and treatment of asthma still remains a major challenge in Africa (16). Distinguishing asthma from other obstructive airway diseases has posed a clinical challenge to clinicians (3,5). Epidemiological data have shown that while asthma presents in episodes, usually among non-smokers and onsets before 40 years, chronic obstructive pulmonary disease (COPD) is associated with smokers, people aged 40 years and above, with symptoms being persistent and progressive (1). In practice, asthmatics who smoke may have non-reversible airflow limitation, and some COPD patients may be non-smokers having reversible airflow limitation (85). In addition, many African countries have no standard protocols for the diagnosis and management of asthma (22); where these are available, guidelines are rarely implemented, and for the few implemented guidelines, treatment often does not reach the rural population that is mostly affected (26). In fact, inaccessibility of health care services in many rural and resource-poor African settings often gives traditional healers undue significance in the management of many chronic diseases (22). The non-availability and unaffordability of inhaled steroids, and the relative non-adherence to these medications (when available) have also had large negative impact on the response to asthma in Africa. Studies have shown that about 50% of people adhere to prescribed medications (26,86), with reasons for non-adherence including side-effects, dosing frequencies, and lack of patient education on their illness, need for treatment, and how to take medications (87). There are also inherent socio-cultural misconceptions and individual values that need to be understood and addressed toward improving the acceptance and use of asthma medications (88), with continual public awareness and education being advocated, especially among parents of children with asthma (5,88).

Asthma is an important and increasing public health problem in Africa which receives inadequate priority and attention. With increasing urbanization, population aging, and adoption of western lifestyles in many African settings, these trends are set to continue in the near future. There is a need to identify and prioritize feasible strategies that can be adopted to promote the implementation of effective interventions that will address this increasing burden in Africa. There is also a need for African national governments to also consider effects of associated risk factors in public health policy planning on this topic with a focus on reducing environmental triggers, placing restrictions on tobacco adverts, and appropriately educating health care personnel and the public on the management of the disease and the preventive measures.
