# Supplementary material for: An estimate of asthma prevalence in Africa: a systematic analysis
Source: Croat Med J. 2013 Dec;54(6):519–31. doi: 10.3325/cmj.2013.54.519 (PMC3893990; doi:10.3325/cmj.2013.54.519)
Supplement: Supplementary Table 1 [file CroatMedJ_54_s016.pdf]

SUPPLEMENTARY ONLINE MATERIAL

Supplementary Table 1. Overall study characteristics

Author	Country	Study Period	Study Setting	Age range/ Mean age	Diagnosis
Ait-Khaled et al. (31)	ISAAC III study (selected countries in Africa)	2000-2003	Mixed	6-7 years; 13-14 years	ISAAC guidelines and scores: based on asthma symptoms (wheeze at rest, wheeze after/during exercise, nocturnal wheeze, nocturnal cough, severe wheeze with speech limitation) using written or video questionnaires
Zar et al. (32)	ISAAC I study (selected countries in Africa)	1994-1995	Mixed	6-7 years; 13-14 years	ISAAC guidelines and scores: based on asthma symptoms (wheeze at rest, wheeze after/during exercise, nocturnal wheeze, nocturnal cough, severe wheeze with speech limitation) using written or video questionnaires
Aguwa et al. (33)	Nigeria	2006	Occupational	>20years	Asthma symptoms, physician's diagnosis, written questionnaire & spirometry (PEFR)
Bennis et al. (34)	Morocco	1991	Rural	< 15 years	Questionnaires based on: "Have you ever had whistling noises in the chest?", "Have you ever had a sensation of respiratory difficulty or suffocation with a whistling noise in the chest?", "Have you ever had asthma?"
Berntsen et al. (35)	Tanzania	2008	Mixed	9-10 years	ISAAC guidelines and scores: based on asthma symptoms (wheeze at rest, wheeze after/during exercise, nocturnal wheeze, nocturnal cough, severe wheeze with speech limitation) using mainly video questionnaires
Bezzaoucha (36)	Algeria	1991	Mixed	<25 years	Asthma symptoms, physician's diagnosis, written questionnaire
Benarab-Boucherit et al. (37)	Algeria	2010	Mixed	10-12 years	6 min free running test (6MFRT), drop in post exercise PEFR >=15% classified as EIB
Desalu et al. (38)	Nigeria	2005-2006	Mixed	32+-10.12 years	ECRHS asthma-screening questionnaire, Subjects with FEV1 <80% of predicted, FEV1/FVC < 80%, or one asthma symptom were subjected to PEF variability testing
Ehrlich et al. (19)	South Africa	1997	Mixed	5-12 years	Asthma symptoms, physician's diagnosis, written questionnaire (filled by parents)
Esamai & Anabwani (39)	Kenya	1995	Rural	13-14 years	ISAAC guidelines, written/video questionnaire

Esamai & Anabwani (40)	Kenya	2001	Rural	13-14 years	ISAAC guidelines, written/video questionnaire
Addo-Yobo et al. (41)	Ghana	1993; 2003	Mixed	9-16 years	6 min free running test (6MFRT), drop in post exercise PEFR ≥12.5% classified as EIB
Dagoye at al. (12).	Ethiopia	2003	Mixed	1-5 years	ISAAC guidelines, physician's diagnosis, written questionnaire
Ehrlich et al. (42)	South Africa	1994	Mixed	>18 years	Asthma symptoms, physician's diagnosis, written questionnaire
Ehrlich et al. (43)	South Africa	2004	Mixed	>18 years	ECRHS guidelines, physician's diagnosis, written questionnaire
Nafti et al. (44)	North Africa-Algeria, Morocco & Tunisia (AIRMAG study)	2008	Mixed	13-14 years; >25 years	GINA criteria "Have you had an asthma attack or asthma symptoms in the previous twelve months? Have you taken medication for asthma attacks (eg pills, inhaled powders, aerosols etc) in the previous twelve months? Have you taken ventolin in the previous twelve months?"
Erharbor et al. (45)	Nigeria	2005	Mixed	15-35 years	IUATLD (1984) & BMRC (1965) criteria
Khaldi et al. (46)	Tunisia	2004	Mixed	13-14 years	ISAAC guidelines, written questionnaire
Koffi et al. (47)	Cote d'Ivoire	1998	Urban	13-14 years	ISAAC guidelines, written questionnaire
Laraqui Hosni et al.(48)	Morocco	2001	Occupational	>20 years	ISAAC guidelines, written questionnaire
Mavale-Manuel et al. (49)	Mozambique	2005	Mixed	13-14 years	ISAAC guidelines, video questionnaire
Miningou et al. (55)	Burkina Faso	1998	Urban	15-64 years	Asthma symptoms, physician's diagnosis, written questionnaire
Falade et al. (50)	Nigeria	1995	Mixed	13-14 years	ISAAC guidelines, written questionnaire
Faniran et al. (51)	Nigeria	1998	Urban	8-11 years	ISAAC guidelines, written questionnaire (modified from Institute of Respiratory Medicine, University of Sydney)
Georgy et al. (52)	Egypt	2005	Mixed	11-15 years	ISAAC guidelines, written questionnaire
Hailu et al. (53)	Ethiopia	1997	Mixed	13-14 years	ISAAC guidelines, written questionnaire
Mashalane et al. (54)	South Africa	2005	Mixed	9-10 years	Free Running Asthma Screening Test (FRAST); drop in post exercise PEFR ≥15% classified as EIB
Mengesha & Bekele (73)	Ethiopia	1997	Occupational	>20 years	Asthma symptoms, physician's diagnosis, written questionnaire
Miszkurka et al. (56)	Burkinafaso	2001	Mixed	>20 years	World Health Survey; Asthma symptoms, physician's diagnosis, written questionnaire

de Almeida et al. (72)	Cape Verde	1993	Mixed	6-10 years	GINA criteria; “Asthma ever was defined as the cumulative lifetime diagnosis. Active asthma identified in children with symptoms during the previous year, and current asthma was considered if a positive bronchial challenge test was found in children with active asthma"
Mustapha et al. (58)	Nigeria	2004	Mixed	7-14 years	Asthma symptoms, physician's diagnosis, written questionnaire
Mtshali & Mokwena (67)	South Africa	2007-2008	Mixed	8-16 years	Free Running Asthma Screening Test (FRAST drop in post exercise PEFR ≥10% on more than 2 occasions classified as EIB
Musafiri et al. (57)	Rwanda	2008-2009	Mixed	≥15 years	ATS criteria; Asthma symptoms, physician's diagnosis, written questionnaire; Subjects who presented with reversible airways obstruction (pre-bronchodilator FEV1/FVC < LLN and a positive reversibility test meaning an increase in FEV1 of 200 ml and 12% with respect to pre-bronchodilator FEV1 after 400 mcg of salbutamol)
Nyembue et al. (61)	DR Congo	2010	Mixed	29±16 years	GINA criteria; Asthma symptoms, physician's diagnosis, skin prick test, written questionnaire
Roudaut et al. (63)	Cote d'Ivoire	1990	Mixed	10-17 years	Asthma symptoms, physician's diagnosis, written questionnaire
Sallaoui et al. (64)	Tunisia	2003	Mixed	20.8±2.7 years	EIB classified as decrease of at least 15% in forced expiratory volume in one second (FEV1) after 8-min running at 80–85%
Terblanche & Stewart (65)	South Africa	1990	Mixed	5-15 years	6MFRT, 10% decline in forced expiratory volume in 1 second after exercise classified as EIB
Wichmann et al. (70)	South Africa	2004-2005	Urban	13-14 years	ISAAC guidelines, asthma symptoms, physician's diagnosis, written questionnaire
Wolff et al. (66)	Madagascar	2010	Urban	7-14 years	Modified ISAAC questionnaire; bronchodilator response (BDR) to establish reversible obstruction (change in FEV1 ≥ 12%)
Ngangan et al. (59)	Kenya	1996	Mixed	10.6 years	6 min free running test (6MFRT), drop in post exercise PEFR ≥15% or >=10% classified as EIB
Nriagu et al. (60)	South Africa	1997-98	Urban	>15 years	Asthma symptoms using standard questionnaire recommended by the World Health Organization for asthma studies

Odhiambo et al. (68)	Kenya	1993	Mixed (Urban/Rural)	10.8 years	IUATLD (1984) guidelines; Asthma symptoms, physician's diagnosis, written questionnaire
Poyser et al. (62)	South Africa	2000	Mixed	13-14 years	ISAAC guidelines, written/video questionnaire
Walraven et al. (69)	Gambia	1996-1997	Mixed (Urban/Rural)	>15 years	IUATLD questionnaire; "Asthma defined by a medical history revealing the periodic occurrence of wheezing and/or (morning) dyspnoea and/or coughing in combination with a decrease of >20% in the FEV1 after provocation with methacholine inhalation at a range of concentrations up to 16 mg/mL, or a 15% increase in FEV1 from baseline 15 min after administering 400 mg inhaled salbutamol. Wheeze was defined as periodic whistling or tightness in the chest. 'Current wheeze' referred to wheezing in the previous 12 months"
Yemaneberhan et al. (71)	Ethiopia	1996	Mixed (Urban/Rural)	22.8 years	IUATLD (1984) questionnaire

6MFRT: 6 minute free running test, ATS: American Thoracic Society, BMRC: British Medical Research Council, EIB: exercise induced bronchospasm, ECRHS: European Community Respiratory Health Survey, FEV1: forced expiratory volume in one second, FRAST: free running asthma screening test, FVC: forced vital capacity, GINA: Global Initiative for Asthma, ISAAC: The International Study of Asthma and Allergies in Childhood, IUATLD: International Union Against Tuberculosis and Lung Diseases, LLN: lower limit of normal, PEFR: peak expiratory flow rate
